# Tobacco taxation: the importance of earmarking the revenue to health care and tobacco control

**DOI:** 10.1186/1617-9625-10-21

**Published:** 2012-12-27

**Authors:** Constantine I Vardavas, Filippos T Filippidis, Israel Agaku, Vasileios Mytaras, Monique Bertic, Gregory N Connolly, Yannis Tountas, Panagiotis Behrakis

**Affiliations:** 1 Center for Global Tobacco Control, Department of Society, Human Development and Health, Harvard School of Public Health, Boston, MA, USA; 2 Smoking and Lung Cancer Research Center, Hellenic Cancer Society, Athens, Greece; 3 Center for Health Services Research, School of Medicine, National and Kapodistrian University of Athens, Athens, Greece

## Abstract

**Background:**

Increases in tobacco taxation are acknowledged to be one of the most effective tobacco control interventions. This study aimed at determining the mediating role of socioeconomical status (SES) and the earmarking of revenue to healthcare and tobacco control, in influencing population support for the adoption of a 2 Euro tobacco tax increase in Greece, amid the challenging economic environment and current austerity measures.

**Methods:**

Data was collected from two national household surveys, the “Hellas Health III” survey, conducted in October 2010 and the "Hellas Tobacco survey” conducted in September 2012. Data was analyzed from 694 and 1066 respondents aged 18 years or more, respectively. Logistic regression models were fitted to measure the adjusted relationship between socio-economic factors for the former, and support for increased taxation on tobacco products for the latter.

**Results:**

In 2012 amidst the Greek financial crisis, population support for a flat two euro tax increase reached 72.1%, if earmarked for health care and tobacco control, a percentage high both among non-smokers (76%) and smokers (64%) alike. On the contrary, when not earmarked, only 43.6% of the population was in support of the equivalent increase. Women were more likely to change their mind and support a flat two-euro increase if the revenue was earmarked for health care and tobacco control (aOR = 1.70; 95% C.I: 1.22-2.38, *p* = 0.002). Furthermore, support for an increase in tobacco taxation was not associated with SES and income.

**Conclusion:**

Despite dire austerity measures in Greece, support for an increase in tobacco taxation was high among both smokers and non-smokers, however, only when specifically earmarked towards health care and tobacco control. This should be taken into account not only in Greece, but within all countries facing social and economic reform.

## Introduction

Smoking is a leading cause of death and disability globally, with significant ramifications on global health and the economy of nations, due to the increased need to cover the economic costs associated with nicotine addiction and its consequences [1,2]. To combat this growing epidemic a plethora of tobacco control activities can be employed, a key initiative of which, is the increase in the price of tobacco products, through taxation. Elevated cigarette prices appear to be associated with a greater motivation to stop smoking [3], while increased prices through tobacco excise taxes are effective in reducing overall tobacco consumption and prevalence [4]. Overall, research has indicated that within developed countries and at a population based level, a 10% increase in the price of cigarettes has been identified to reduce overall cigarette consumption by 2.5% to 5.0% [5], while further evidence from low and middle income countries suggest that even larger reductions in consumption are obtainable [6]. While increases in the price of tobacco products are effective in reducing consumption and prevalence across age groups and social strata, research has indicated that the greatest reductions are noted among participants of lower socio-economic status (SES) [2].

With the above in mind, our aim was to 1) assess population support for an increase in tobacco taxation in Greece during the period of 2010 to 2012, 2) to investigate determinants of support and to test the hypothesis that socio-economic status might act as a significant predictor of support for policies that propose to increase tobacco taxation. Finally, 3) assess the population factors that influence the change of opinion to support a tax increase, if the revenue is earmarked for tobacco control activities and health care, in comparison when this revenue is not earmarked.

## Methods

Data was collected through two nationwide surveys: the Hellas Health III Survey in 2010 and the Hellas Tobacco Survey, in 2012, herein referred to as the National Survey 2010 and 2012, respectively).

### Hellas health III survey (National Survey, 2010)

The national household survey “Hellas Health III” was conducted in Greece in October, 2010. The designed survey sample consisted of 1,000 individuals, aged 18 years or more, that lived in urban (2,000 or more inhabitants) and rural areas (less than 2,000 inhabitants) of the country and in each of the 13 geographical regions. Respondents were selected by means of a three stage, proportional to size, sampling design. At the first stage, a random sample of building blocks was selected proportionally to size based on the 2001 Population Census. At the second stage, in each selected area of blocks, the households to be interviewed were randomly selected by means of systematic sampling. Any person or group of persons living in a separate housing unit was considered as a ‘household’ unit. At the third stage, in each household, a sample of individuals 18 years old or more was selected by means of simple random sampling [7]. The sample was representative of the Greek population in terms of age and residency. The final outcome -support for an increase in tobacco taxation- was measured in 694 respondents (out of the initial 1000 individuals) with 306 missing observations, due to the positioning of the specific subset of questions within the questionnaire (located in the final section). However, the final respondents’ age, gender, residence or socioeconomic status did not differ to that of the entire population.

Participants were classified as current cigarette smokers if they indicated that they smoked any cigarettes (including hand-rolled and other forms of tobacco products), any day during the past month. Respondents were requested to note their support for a potential increase in tobacco taxation, through the question “Are you in favor of an increase in tobacco taxation?”, to which respondents could answer either: yes, probably yes, probably no and no. Positive (supportive) responses were grouped together (yes and probably yes), as were negative (no, probably no). Within the 2010 survey, SES was defined using a 3-point scale (high, middle and low SES) adapted from the 8-point ESOMAR social grade index based on the combination of the occupation and terminal education of the main income earner of the family. Based on the above, the respondents were classified into one of the following categories: A, B, C1, C2, D, E1, E2, E3. Participants in this study were grouped as high (A,B) vs. middle (C1/C2), vs. low (D/E responses), and high vs. middle/low.

### Hellas tobacco survey (National Survey, 2012)

The Hellas Tobacco Survey was conducted via phone in October 2012. The sample was representative to the population of Greece in terms of residence, and consisted of 1066 individuals aged 18 years or more. Participants were classified as smokers if they responded that they smoked any day during the past month. Income level was assessed with the question "with your current income, would you say that you make ends meet very easily, easily, difficultly or very difficultly". People who responded “easily” and “very easily” were considered to be in the higher income group, those who responded "difficulty" and "very difficultly" in the lower income group. Support for cigarette tax increases was assessed with two questions; "do you support an increase of tobacco tax by two euros?" and the second one was "do you support an increase of tobacco tax by two euros, if the revenue were used to improve health care and to prevent youth from smoking?". Individuals who would not support a tax increase unless the revenue were used to improve health care and to prevent youth from smoking, were also identified.

### Statistical analysis

Bivariate analyses were conducted using the chi squared test to determine the unadjusted relationship between support for an increase in tobacco taxation (for the 2010 survey) or for support of a two euro tax increase (for the 2012 survey) and covariates. For the 2010 National Survey data, variables that attained a significance level of *p* = 0.2 in the bivariate analysis were included in the final model, and included age and current smoking status. Also, other socio-demographic variables were included apriori into the model (residence, gender). Finally, logistic regression analyses were performed to determine the adjusted relationship between the outcomes of interest and the covariates, adjusting for age, gender and residence. For the 2012 National Survey data, three different logistic regression analyses were performed, one for each of the following variables: support of a two-euro tax increase, support of a two-euro tax increase if the revenue were used to improve health care and to prevent youth from smoking and support of a two-euro tax increase only if the revenue were earmarked but not if it weren't. Age, income, education, gender and current smoking status were included in the logistic regression models. Data analysis was performed with SPSS.V17.

## Results

### National survey 2010

In 2010, almost 1 in 2 respondents (46%, n = 321) supported an increase in the taxation on tobacco products, however among non-smokers, support reached 67.1%, in contrast to only 20.4% of current smokers. (*p* < 0.001). Within the bivariate analysis, age was also strongly associated with support for increased taxes on tobacco products (49.4% of older respondents vs. 38.7% of younger respondents, *p* = 0.001). When examining support for increased taxes by SES, this was not found to significantly differ by SES, as 50.6% of high vs. 59.2% of middle vs. 44.9% of low SES respondents supported an increase in taxation (*p* = 0.103). Separating SES by smoking status, there was still no difference in support between participants of different SES (*p* = 0.856). After adjusting for age, gender, current smoking status and place of residence, SES was still not a significant predictor of support for an increase in tobacco taxation (aOR: 0.68; 95%C.I: 0.37-1.25, *p* = 0.211). As seen in Table 1, within the multivariate analysis, the strongest predictor of non-support for increased taxation on tobacco products was current smoking status (aOR: 0.12; 95% C.I: 0.08-0.17 *p* < 0.001). While a younger age was identified as a significant mediator in the crude analysis, this disappeared in the adjusted (aOR: 0.69; 95% C.I: 0.47-1.01, *p* = 0.059). Neither gender (*p* = 0.366) nor residence (*p* = 0.560) were significantly associated with support for an increase in tobacco taxation.

**Table 1:** Predictors of support for a non-earmarked increase in tobacco tax in Greece, October 2010

		**Adjusted**^**1**^		**Crude**		
**Variable**	**aOR**	**95% C.I**	***p- *Value**	**OR**	**95% CI**	***p*- Value**
Socioeconomic status	0.68	0.37 - 1.25	0.211	0.71	0.42 - 1.21	0.205
Age	0.69	0.47 - 1.01	0.059	0.61	0.43 - 0.85	0.003
Gender	1.17	0.83 - 1.66	0.366	1.15	0.85- 1.56	0.373
Current Smoking status	0.12	0.08 - 0.17	<0.001	0.14	0.09- 0.19	<0.001
Residence	0.89	0.60 - 1.31	0.560	0.94	0.67- 1.33	0.741

1: Logistic regression model adjusted for age (≤ 34 years vs. >34 years*), SES (High vs. other*), gender (male* vs. female), residence (urban* vs. rural), current smoking status (current smoker vs. otherwise*). Asterisks (*) indicate reference groups.

### National survey 2012

**Figure 1:**
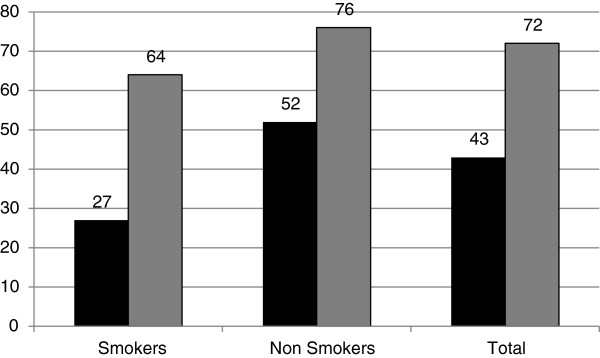
Population support for an earmarked (grey column) and non-earmarked (black) increase in tobacco taxation by 2 Euro, by smoking status, Greece 2012.

In 2012, two years after the 2010 survey, support for a flat two euro tax increase was estimated at 43.6%, however, this percentage greatly increased to 72,1%, if the two euro tax increase was to be earmarked for health care and tobacco control. Smokers were again less likely to support the tax increase in both cases (aOR: 0.27 and 0.46 respectively, p < 0.001), however the percentage of support still remained substantially elevated. (Figure 1) Within a regression analysis and when exploring the characteristics of the individuals who would only support the tax increase if the revenue was earmarked, only the participants gender was found to be a statistically significant factor, as 58.2% of the women who would not support a two-euro tax increase would change their mind if the revenue were earmarked versus 46.7% of men (p < 0.01). The respondents income (aOR: 0.88; 95% C.I: 0.52-1.48, *p* = 0.627) and education (aOR: 1.15; 95% C.I: 0.80-1.65, *p* = 0.463) were not statistically associated with changing views in regards to a tax increase in the multivariate model, whereas gender remained a statistically significant predictor (aOR: 1.70; 95% C.I: 1.22-2.38, *p* = 0.002). Income, age and education were not statistically significant factors in any of the logistic regression models (Tables 2 and 3).

**Table 2:** Characteristics of the population in support of a 2-euro tobacco tax increase if funds are earmarked for health care and tobacco control, and those in support if it is not earmarked, in September 2012, Greece

		**Adjusted**^**1**^		**Crude**		
**Variable**	**aOR**	**95% C.I**	***p- *Value**	**OR**	**95% CI**	***p- *Value**
**Factors related to support for a 2 Euro tax increase if the revenue is not earmarked**^**2**^						
Income	1.17	0.74 - 1.87	0.504	1.21	0.76 - 1.91	0.423
Age	0.83	0.56 - 1.24	0.369	0.83	0.56 - 1.23	0.356
Gender	1.39	1.03 - 1.88	0.031	1.43	1.07 - 1.92	0.016
Current Smoking status	0.46	0.34 - 0.62	<0.001	0.44	0.33 - 0.60	<0.001
Education	1.12	0.81 - 1.56	0.485	1.11	0.81 - 1.52	0.525
**Factors related to support of a 2 Euro tax increase If the revenue is earmarked**^**3**^						
Income	1.12	0.75 - 1.67	0.577	1.25	0.86 - 1.83	0.251
Age	0.78	0.54 - 1.14	0.198	0.80	0.56 - 1.13	0.201
Gender	0.93	0.71 - 1.21	0.577	1.00	0.77 - 1.28	0.972
Current Smoking status	0.27	0.20 - 0.36	<0.001	0.27	0.21 - 0.36	<0.001
Education	1.18	0.88 - 1.57	0.272	1.17	0.89 - 1.53	0.266

1: Logistic regression models adjusted for age (≤ 34 years vs. >34 years*), Income (High vs. middle/low*), gender (male* vs. female), education (university vs. other*), current smoking status (current smoker vs. otherwise*). Asterisks (*) indicate reference groups.

2: These respondents answered yes, when asked if in favor of a 2 Euro tobacco tax increase.

3: These respondents answered yes, when asked if in favor of a 2 Euro tobacco tax increase earmarked towards health care and tobacco control.

**Table 3:** Characteristics of the population^1^ that support a two Euro tobacco tax increase only if the revenue is earmarked towards health care and tobacco control

		**Adjusted**^**2**^		**Crude**		
**Variable**	**aOR**	**95% C.I**	***p- *Value**	**OR**	**95% CI**	***p- *Value**
Income	0.88	0.52 - 1.48	0.627	0.81	0.49 - 1.36	0.432
Age	1.09	0.69 - 1.72	0.714	1.10	0.70 - 1.71	0.686
Gender	1.70	1.22 - 2.38	0.002	1.58	1.15 - 2.19	0.005
Current Smoking status	0.95	0.68 - 1.33	0.775	0.93	0.67 - 1.28	0.637
Education	1.15	0.80 - 1.65	0.463	1.19	0.83 - 1.69	0.345

1: These respondents answered no, when asked if in favor of a 2 Euro tobacco tax increase, and yes, if this increase is earmarked towards health care and tobacco control.

2: Logistic regression models adjusted for age (≤ 34 years vs. >34 years*), Income (High vs. middle/low*), gender (male* vs. female), education (university vs. other*), current smoking status (current smoker vs. otherwise*). Asterisks (*) indicate reference groups.

## Discussion

Based on our findings, the majority of the Greek population was in support of a flat 2 Euro increase in tobacco taxation, despite the dire financial and social environment in Greece in 2012. This support was not associated with the respondents SES or income, but only with their current smoking status and age. When earmarking the revenue, support for an increase in tobacco taxation was noticed, with women found to be more supportive than men, and more likely to change their mind and support the proposed policy. This support was noted even when taking current smoking into account. This suggests that more disadvantaged and more affluent smokers are equally engaged with tobacco control objectives, which is also supported by the absence of a social gradient in rates of stop-smoking attempts in England [8,9]. Additionally, price increases are known to possibly have a disproportionate financial impact on low-income smokers, who benefit the most from such incentives [10]. Research has also indicated that smokers of a lower SES were about 25% more likely to utilize at least one price or tax avoidance strategy during their last purchase and have been noted to be more likely to switch to either roll-your-own tobacco or discounted brands to reduce the cost of their habit [11]. Indeed under the current taxation strategy in Greece, within which roll-your-own tobacco is under taxed in comparison to manufactured, consumer switching, might be an issue. The equalizing of tax rates for different tobacco products in Greece could potentially eliminate this loophole, and should be pursued.

The WHO Framework Convention on Tobacco Control (FCTC) calls for parties to use tobacco pricing and taxes as an effective way of reducing tobacco consumption, an activity which may be subject to industry manipulation [12,13]. This has been demonstrated to be an effective strategy in preventing initiation and uptake among young users, promoting cessation among current users and reducing consumption among those who continue to use [2]. When not earmarked, slightly less than half of the population supported the proposed increase in tobacco taxation, which is very similar to the percentage of the UK population which was noted to be in support of an increase in tobacco taxation (48%), on the contrary, when earmarked for health care and tobacco control, 72% of the respondents in Greece supported the increase. This is an important fact, as tobacco taxation is an important activity in preventing tobacco use among youth also [14]. This response is of special importance for Greece, since over the past 5 years, the national deficit has risen dramatically, with severe implications for the already ailing Greek health care system [15].

While the two nationwide studies are representative of the Greek population regarding the place of residence, they can only indicate associations. While the results are indicative, a larger sample would have been needed to assess with detail the role of socio-economic factors in mediating support for an increase in tobacco taxation. Moreover, the current analyses handled SES and income as bivariate variables, and thus we were unable due to sample size, to perform a larger more detailed analysis taking each stratum into account. Further research on this topic in Greece is needed and is currently being planned. Moreover, the slightly different design of the two studies (in person vs. telephone) do not allow direct comparisons between them, thus we have limited our analysis and interpretation to analyzing each study individually.

As Greece struggles with the current financial strains and austerity measures, it is noteworthy that a flat two Euro increase in tobacco taxation is supported by the majority of the population -smokers and non-smokers alike- if the revenue is earmarked towards the ailing health care system and tobacco control. Earmarking of the anticipated revenue thus could be employed as an effective way to earn public's support, and to provide funding tobacco control related activities.

## Competing interests

The authors’ declare that they have no competing interests.

## Author contributions

Authors CIV, FF, IA and VM, had the main role in manuscript preparation, data interpretation and data analysis, with authors MB, GNC, YT and PB actively participating in study design, data collection, data interpretation and manuscript preparation also. All authors read and approved the final manuscript.
